# Missense *NAA20* variants impairing the NatB protein N-terminal acetyltransferase cause autosomal recessive developmental delay, intellectual disability, and microcephaly

**DOI:** 10.1038/s41436-021-01264-0

**Published:** 2021-07-06

**Authors:** Jennifer Morrison, Norah K. Altuwaijri, Kirsten Brønstad, Henriette Aksnes, Hessa S. Alsaif, Anthony Evans, Mais Hashem, Patricia G. Wheeler, Bryn D. Webb, Fowzan S. Alkuraya, Thomas Arnesen

**Affiliations:** 1 Division of Genetics, Arnold Palmer Hospital, Orlando, FL USA; 2 Department of Translational Genomics, Center for Genomic Medicine, King Faisal Specialist Hospital and Research Center, Riyadh, Saudi Arabia; 3 Department of Biomedicine, University of Bergen, Bergen, Norway; 4 Department of Genetics and Genomic Sciences, Icahn School of Medicine at Mount Sinai, New York, NY USA; 5 Department of Biological Sciences, University of Bergen, Bergen, Norway; 6 Department of Surgery, Haukeland University Hospital, Bergen, Norway

## Abstract

**Purpose:**

N-terminal acetyltransferases modify proteins by adding an acetyl moiety to the first amino acid and are vital for protein and cell function. The NatB complex acetylates 20% of the human proteome and is composed of the catalytic subunit NAA20 and the auxiliary subunit NAA25. In five individuals with overlapping phenotypes, we identified recessive homozygous missense variants in *NAA20*.

**Methods:**

Two different *NAA20* variants were identified in affected individuals in two consanguineous families by exome and genome sequencing. Biochemical studies were employed to assess the impact of the *NAA20* variants on NatB complex formation and catalytic activity.

**Results:**

Two homozygous variants, *NAA20* p.Met54Val and p.Ala80Val (GenBank: NM_016100.4, c.160A>G and c.239C>T), segregated with affected individuals in two unrelated families presenting with developmental delay, intellectual disability, and microcephaly. Both NAA20-M54V and NAA20-A80V were impaired in their capacity to form a NatB complex with NAA25, and in vitro acetylation assays revealed reduced catalytic activities toward different NatB substrates. Thus, both NAA20 variants are impaired in their ability to perform cellular NatB-mediated N-terminal acetylation.

**Conclusion:**

We present here a report of pathogenic *NAA20* variants causing human disease and data supporting an essential role for NatB-mediated N-terminal acetylation in human development and physiology.

**Graphical Abstract:**

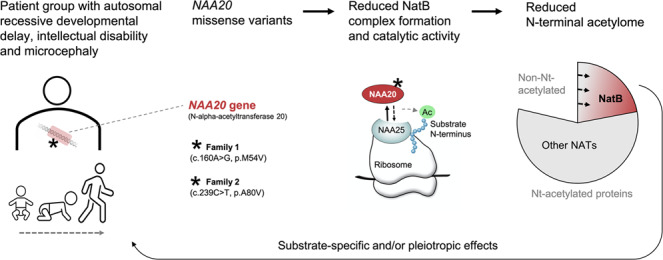

## INTRODUCTION

N-terminal acetylation is a common protein modification in eukaryotes, and approximately 80% of all human proteins carry this modification [1, 2]. Although not fully understood, N-terminal acetylation may have a range of functional consequences for the modified proteins including stability/degradation, subcellular targeting, and complex formation [1]. NatB is one of the major eukaryotic N-terminal acetyltransferases (NATs) acetylating around 20% of the human proteome in a cotranslational manner. Proteins harboring Met-Glu-, Met-Asp-, Met-Gln-, and Met-Asn-N-termini are substrates of NatB [3]. The catalytic subunit NAA20 forms a stable heterodimer with the large ribosomal anchor subunit NAA25 [4, 5]. NatB activity has been linked to cancer cell survival and progression [4, 6, 7] as well as shutoff activity of influenza A virus and viral polymerase activity [8] and NAD^+^/NADH metabolism [9]. However, no genetic disease has so far been linked to pathogenic variants of the *NAA20* or *NAA25* genes.

We report here five affected individuals of two unrelated families presenting with developmental delay (DD), intellectual disability (ID), and microcephaly. Homozygous *NAA20* variants (MIM 610833) segregated with the phenotypes. Protein studies revealed impaired functionality of both identified variants supporting that reduced cellular N-terminal acetylation is causative for disease.

## MATERIALS AND METHODS

*NAA20* variants were discovered through exome or genome sequencing after clinical evaluation. Contact between clinicians and researchers was mediated by GeneDx/GeneMatcher [10]. For further experimental details, see Supplemental Materials and Methods.

## RESULTS

### Genetic findings

**Fig. 1:**
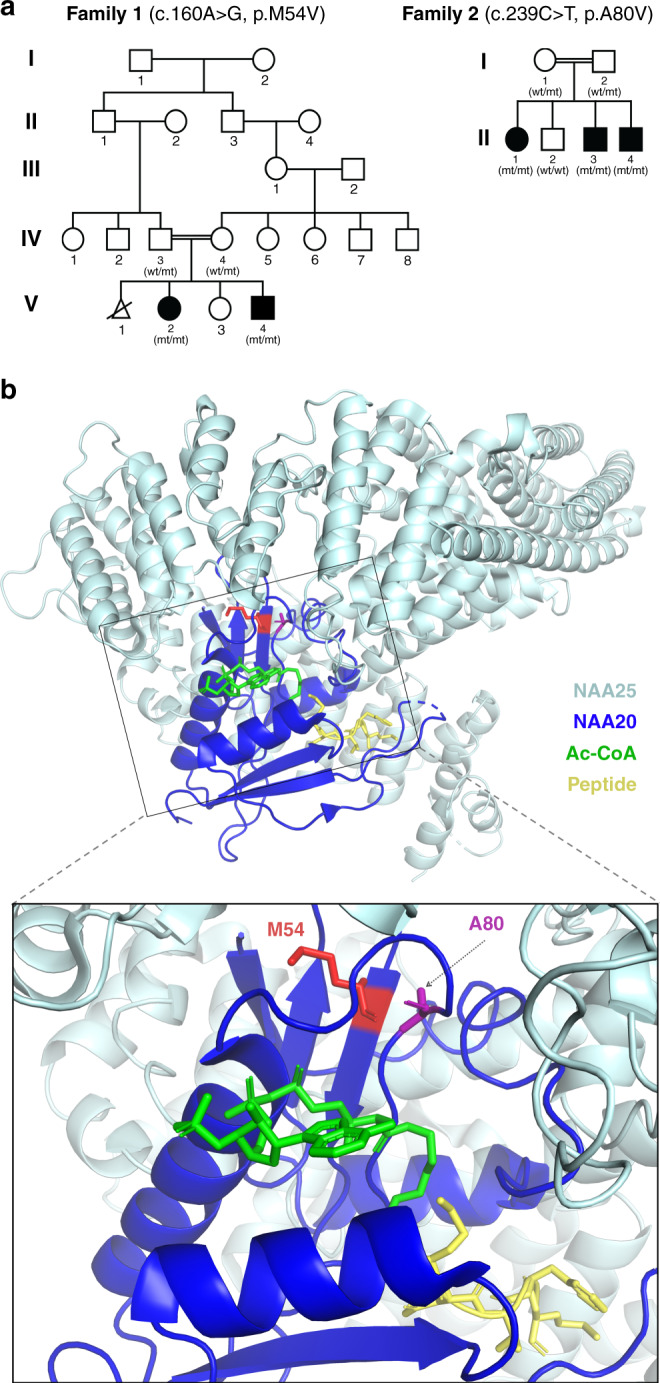
Rare *NAA20* variants segregating with developmental phenotypes in two families. (**a**) Pedigrees of family 1 and family 2 with affected members indicated as filled circles (females) or squares (males). Triangle: pregnancy not carried to term. Double horizontal lines indicate consanguinity. Wt and mut indicate absence or presence of the *NAA20* variants, respectively. (**b**) Three-dimensional structure model of NAA20 and NAA25 visualizing the positions of the variant sites in the wildtype NAA20 structure. Gray, NAA25; blue, NAA20; green, Ac-CoA; yellow, substrate peptide.

The index case in family 1, a 13-year-old female (F1:V.2) (Fig. 1a) of Saudi origin, was referred for neuropsychological evaluation for baseline cognitive assessment because of her global DD and significant ID. She is the eldest of three siblings, with a healthy sister and a brother similarly suffering from DD and ID (F1:V.4). Parents are both healthy and are paternal cousins. Exome sequencing performed on DNA from the two affected siblings uncovered a homozygous missense variant of uncertain significance in *NAA20* (NM_016100.5): c.160A>G (p.Met54Val) (GenBank: NM_016100.4). We employed both positional mapping to highlight candidate common regions within the genomes of the affected and exome sequencing to identify the most likely candidate variant(s) within these critical loci. Upon analyzing the family’s genotyping data, we identified eight regions of homozygosity (ROHs) that were exclusively shared between the two affected siblings. We prioritized novel/rare (minor allele frequency [MAF] < 0.001 based on gnomAD and 2,379 local exomes), homozygous, coding/splicing variants within these regions that minimized the search to the single *NAA20* variant. Segregation analysis of the variant confirmed that both parents were heterozygous, whereas both affected siblings were homozygous (Fig. S1). The in silico prediction of this variant suggests its likely deleterious nature using BayesDel_addAF, CADD, FATHMM-MKL, LIST-S2, MutationTaster, and PrimateAI (Table S1).

In family 2, three affected siblings (F2:II.1, F2:II.3, F2:II.4) of Iraqi origin who were born to consanguineous parents presented for clinical genetics evaluation due to history of DD and microcephaly (Fig. 1a). Chromosomal microarray for the three affected siblings revealed common areas of absence of heterozygosity (AOH) at hg19 coordinates chr20:10,418,800-16,923,134 and chr20:29,448,795-41,483,591. Genome sequencing identified a homozygous missense variant of unknown significance in *NAA20* (c.239C>T [p.Ala80Val] [GenBank: NM_016100.4]) in the three affected siblings (Fig. S1; Supplemental Materials and methods).

The *NAA20* c.239C>T (p.Ala80Val) variant is predicted to be deleterious using BayesDel_addAF, CADD, DANN, EIGEN, FATHMM-MKL, LIST-S2, MutationTaster, PrimateAI, and SIFT (Table S1). Neither of these two *NAA20* variants were contained in gnomAD.

### Clinical findings

The clinical findings of the five affected individuals are partially overlapping and are summarized in Table S2 and in the Supplemental case reports.

DD was present in all affected individuals. Head circumference was reduced, with microcephaly (between -2.3 SD and -3.5 SD) for four individuals and borderline microcephaly for F1:V.4 (-1.9 SD). Ability to walk was delayed and observed at 2–3.5 years. All individuals have a limited ability to speak. The female proband of family 2 (F2:II.1) only uses a few words appropriately at age 10 years. Vision and hearing appear to be normal for all. Some variable dysmorphic features are observed for 4/5 individuals, such as prominent philtrum, thick upper lips and epicanthal folds, downslanting of palpebral fissures, and wide-spaced teeth. Mild to moderate ID is observed for all cases, and autistic features are noted for 2/5 individuals (one in each family). For all three affected individuals in family 2, but none of the affected individuals in family 1, cardiac anomalies were observed. F2:II.1 and F2:II.3 have ventricular septal defects, while F2:II.4 has patent ductus arteriosus.

### Functional analysis of *NAA20* variants

To define whether and how these two *NAA20* variants impair NAA20 protein function, we further investigated their structural and biochemical properties. Both NAA20 Met54 and Ala80 are evolutionarily conserved residues in many eukaryotic species suggesting functional importance (Fig. S2). Met54 and Ala80 are structurally positioned in the vicinity of NAA25, the binding partner of NAA20 in the functionally active NatB complex (Fig. 1b). Thus, it is possible that altering these residues may impact the ability of NAA20 to bind NAA25.

**Fig. 2:**
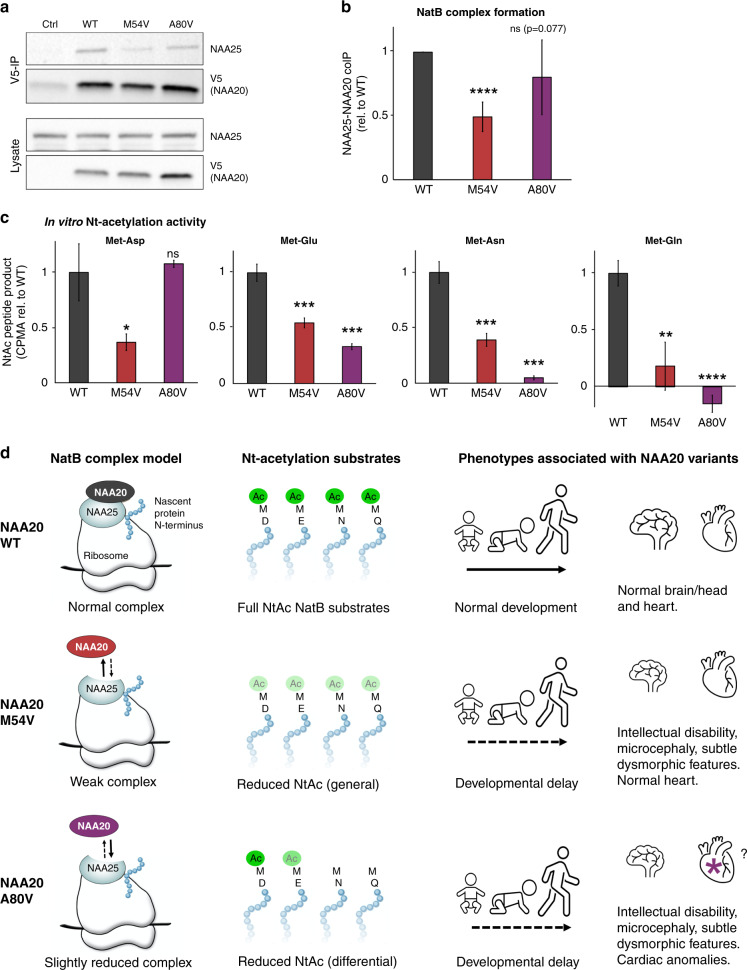
NAA20-M54V and A80V have impaired capacity to form NatB complexes and to catalyze NatB-mediated N-terminal acetylation. (**a**) HeLa cells were transfected with Ctrl-V5 and NAA20-V5 constructs, lysed, and immunoprecipitated with anti-V5. Lysate (lower) and immunoprecipitation (IP) samples (upper) were immunoblotted with anti-V5 and anti-NAA25. Image shown is the representative result of nine independent experiments. (**b**) Quantification of NatB complex formation based on immunoprecipitation experiments as shown in (**a**) (*n* = 9). Ratio NAA25 immunoprecipitated by NAA20. Data are presented as mean +/− s.d. ****p < 0.00005 by two-tailed *t*-test with unequal variance. (**c**) NAA20-V5 wild type (WT) or variants were expressed in HeLa cells and isolated by IP. IP product was used as input in N-terminal acetylation assays using synthetic peptides representing one of four NatB type substrates (Met-Asp, Met-Glu, Met-Asn, Met-Gln) and [14 C]-Acetyl Coenzyme A. Data from three independent experiments were pooled. Reaction mix with control (Ctrl) IP products served as blank and was subtracted. V5-control plasmid was used for negative Ctrl. Values are corrected for immunoblot band intensity and expressed as relative to the WT. Data are presented as mean +/− s.d. Error bars show standard deviation. *p < 0.05; **p < 0.005; ***p < 0.0005; ****p < 0.00005 by two-tailed *t-*test with unequal variance. (**d**) Schematic model of *NAA20*-related syndrome. At the molecular level, NAA20-M54V weakly associates with NAA25 while NAA20-A80V is only moderately impaired in NatB complex formation. The formed NatB complexes of NAA20-M54V are additionally impaired in catalyzing N-terminal acetylation of all NatB type substrates, while NatB complexes of NAA20-A80V display normal activity toward Met-Asp N-termini and impaired activity toward Met-Glu, and in particular Met-Asn, and Met-Gln N-termini. The decreased capacity to acetylate various N-termini of cellular proteins has diverse pathophysiological effects such as developmental delay, intellectual disability, and microcephaly. For NAA20-A80V cases, cardiac anomalies are also observed, but identification of further individuals is required to define this as a phenotype typical for NAA20-related syndrome or a specific subgroup defined by specific substrate targeting.

To investigate the potential impact of the variants on NatB complex formation, NAA20-WT-V5, or variants were immunoprecipitated from HeLa cells. Western blotting analysis revealed that both NAA20-M54V and NAA20-A80V coimmunoprecipitated less NAA25 as compared to NAA20-WT (Fig. 2a, b). The defect of NAA20-M54V was consistently more severe than that of NAA20-A80V. We found no difference in the cellular stability of the two NAA20 variants as compared to NAA20-WT by cycloheximide chase assay (Fig. S3). This was further supported by the fact that NAA20 levels were unchanged in lymphoblasts in all affected individuals of family 2 as compared to control lymphoblasts (Fig. S4). Importantly, we defined the intrinsic catalytic N-terminal acetyltransferase activity of the two variants in vitro (Fig. 2c). We here assessed the activity toward peptides representing all four types of NatB substrates, Met-Glu-, Met-Asp-, Met-Gln-, and Met-Asn-. While NAA20-M54V exhibited a reduced NatB activity toward all four substrate classes, NAA20-A80V displayed alterations in a substrate-specific manner. NAA20-A80V was not reduced in its capacity to acetylate a Met-Asp substrate, but it revealed a significant loss in its capacity to acetylate Met-Glu, Met-Asn, and Met-Gln substrates (Fig. 2c). Since the catalytic subunit NAA20 depends on complex formation with NAA25 to form the active NatB complex on the ribosome, impaired binding between NAA20 and NAA25 will result in less active NatB complexes capable of modifying nascent polypeptides, including those starting with Met-Asp. In addition, the decreased intrinsic activities of NAA20-M54V and NAA20-A80V will further reduce the cellular N-terminal acetylation of many NatB substrates. In sum, both NAA20 variants are less competent than NAA20-WT in performing cellular NatB-mediated N-terminal acetylation of Met-Glu-, Met-Asp-, Met-Gln-, and Met-Asn-N-termini.

## DISCUSSION

Based on our functional studies, it is highly likely that the individuals homozygous for the *NAA20* c.160A>G (p.Met54Val) or *NAA20* c.239C>T (p.Ala80Val) variants suffer from impaired NatB-mediated N-terminal acetylation of numerous cellular substrates. Because there are several thousand different NatB substrates in human cells [3] and because NatB steers many cellular pathways [1], pathogenic *NAA20* variants are likely to have pleiotropic effects. This fits well with the overall findings of DD and ID in all individuals. However, NAA20-M54V and NAA20-A80V displayed differences in their substrate specificities, with NAA20-M54V relatively more impaired in its ability to acetylate Met-Asp substrates while NAA20-A80V was comparatively less active toward the other substrate types (Fig. 2c). This might suggest that there are also certain cellular NatB substrates that are specifically impacted for each of these two *NAA20* variants. Thus, unique clinical findings for affected individuals harboring a specific *NAA20* variant may relate to disrupted signaling via specific NatB substrates only impaired for a specific variant (Fig. 2d). For example, only affected family 2 individuals, not affected family 1 individuals, presented with cardiac anomalies (Table S2). However, more individuals need to be identified to properly define the genotype–phenotype relationship, and differences in genetic background between individuals may significantly contribute to observed phenotypic differences.

In humans, seven distinct NAT enzymes (NatA–NatF and NatH) have been identified [1]. Each NAT is composed of unique subunits and catalyzes N-terminal acetylation of a unique set of substrates. NatA–NatE perform cotranslational N-terminal acetylation. While NatA, NatB, and NatC perform bulk acetylation of large substrate pools, NatD and NatE have more specialized roles toward a few substrates. In contrast, NatF and NatH act post-translationally toward transmembrane proteins and actins, respectively [1].

Until now, pathogenic variants were only identified for genes encoding the catalytic NAA10 and auxiliary NAA15 subunits of the NatA complex. In 2011, the lethal X-linked Ogden syndrome was presented. Eight boys harboring a *NAA10* missense variant displayed an aged appearance, craniofacial anomalies, hypotonia, global DD, cryptorchidism, and cardiac arrhythmias [11]. Investigations in budding yeast and patient cells suggested that a reduced NatA mediated N-terminal acetylation was involved in disease etiology [12–14]. In the last decade, a number of additional pathogenic *NAA10* variants were identified in boys and girls presenting with ID, DD, and cardiac abnormalities [15–17]. Distinct phenotypes such as Lenz microphthalmia syndrome (MIM 309800) were also correlated to specific effects of some variants. The potential multifunctionality of NAA10 as a monomeric NAT and KAT in addition to its role as a catalytic subunit of the NatA complex (together with NAA15) makes it very challenging to define disease mechanisms [1], although some variants are more impaired in NatA function while others are more impaired in monomeric NAA10 function. More recently, patients harboring pathogenic *NAA15* variants also presented with phenotypes partially overlapping with those observed for *NAA10* variants, including cohorts of patients with congenital heart disease and autism spectrum disorder [17–20]. Thus, it is likely that impaired NatA mediated N-terminal acetylation is at least in part causative for disease seen in these individuals. Despite the fact that NatA and NatB acetylate unique subsets of cellular substrates, at present, it is difficult to distinguish between NatA and NatB-mediated impairment of N-terminal acetylation at the level of human pathophysiology. This is due to the pleiotropic nature of overlapping phenotypes as well as extensive phenotype variability among individuals with pathogenic *NAA10*, *NAA15*, and *NAA20* variants. Microcephaly is potentially a distinguishing parameter that is only found in some *NAA10* and *NAA15* variant cases [17], but was found in this study among all affected individuals with *NAA20* variants (Table S2). Unlike *NAA10* and *NAA15*, *NAA20* appears to be more tolerant to haploinsufficiency (probability of loss of function intolerance [pLI] = 0.01) and less constrained for missense variation (*Z* = 0.31). These characteristics are consistent with the strictly recessive inheritance of the variants we report in this study in *NAA20* in contrast to the monoallelic disease-causing variants reported previously in *NAA10* and *NAA15*.

In conclusion, we present here pathogenic *NAA20* variants that disrupt NAA20 function and support an essential role for NatB-mediated N-terminal acetylation in human development and physiology. All affected individuals display DD, ID, and microcephaly. We propose to use the term *NAA20*-related syndrome to describe this novel disorder caused by pathogenic *NAA20* variants.

## Supplementary information

Supplementary Information

Supplementary Table 3

## Data Availability

The *NAA20* variants with accession numbers are available at LOVD: https://databases.lovd.nl/shared/variants/0000763620#00014229 and https://databases.lovd.nl/shared/variants/0000763619#00014229.
