# Supplementary material for: Missense NAA20 variants impairing the NatB protein N-terminal acetyltransferase cause autosomal recessive developmental delay, intellectual disability, and microcephaly
Source: Genet Med. 2021 Jul 6;23(11):2213–8. doi: 10.1038/s41436-021-01264-0 (PMC8553619; doi:10.1038/s41436-021-01264-0)
Supplement: Supplementary file 1 — Supplementary Information [file 41436_2021_1264_MOESM1_ESM.docx]

**Supplemental case reports**

**Family 1:**

Parents are both healthy and are paternal cousins (1st cousins once removed). The index (F1:V.2) was 11 years old at the time of recruitment with a similarly affected younger brother (F1:V.4) who was recruited at 6 years of age. The index was referred for neuropsychological evaluation for baseline cognitive assessment because of her global developmental delay and significant intellectual disability. She was noted to have delayed development at around the age of 6-12 months. The index (F1:V.2) is the eldest of three siblings; her sister (age 7) (F1:V.3) is healthy, but her brother (F1:V.4) suffers from developmental delay (see below). She is a product of full-term pregnancy and cesarean delivery due to cephalo-pelvic disproportion. At age six months, she suffered a head injury with no loss of consciousness, followed by staring and lethargy. However, the parents noted that her developmental delays were noticed even before the head injury. There is no overt history of seizures.

Developmentally, the index achieved motor and language milestones moderately late (e.g. sat at age 1:0 year; walked at age 2:6 years; several words by age 5:0 years). She speaks like a 2-year-old her mother notes. She was toilet trained at age five. At five years old, she was enrolled in a rehabilitative center where she attended sessions for speech and occupational therapy for 45-minutes a day. The next year, she attended the full-time day program at the same center. Her parents note that she "understands well," is able to request things, but does not organize her speech or articulate as expected. She memorizes nursery rhymes. Her parents note a generally stable mood, but frequent bouts of irritability when she fails to socially mingle with her same-age cousins.

Index’s medical assessment:

Medical examination (2014) revealed a head circumference (48.5 cm) at the 10th percentile, her weight and height were at 75th percentile, and subtle dysmorphic features in the form of prominent philtrum with thick upper lips and epicanthal folds. MRI brain study revealed patchy foci of increasing signal intensity seen in the bilateral frontal subcortical region. Chest, cardiovascular, abdominal, and CNS examinations were normal. Her routine labs including CBC, renal and liver function tests, were normal. Her creatine metabolites, CDT, tandem metabolic screen, urine organic acids, all within normal limits. Her liver function test and creatine kinase were also normal. A creatine panel to search for creatine disorders as a cause of intellectual disability was done but the panel was normal. Her skeletal survey showed generalized osteopenia, mild scoliosis and right little finger clinodactyly but otherwise normal.

Neurocognitive exam and behavioral observations:

She presented as a pleasant neatly groomed girl. Subtle dysmorphic autistic features were noted. Gait and overall motor function were unremarkable. She was able to list the names of her siblings, mostly in the correct order, but her speech was generally limited for her age and poorly articulated (letter substitution). She smiled appropriately according to social context and made good eye contact. She often awaited affirmation from examiner’s facial expressions. Halfway through testing, she seemed to have lost interest and did not participate. A reward chart and encouragement were presented, which significantly increased her motivation. Mild restlessness was noted, especially toward the end. Overall, she attempted every task presented to her, maintained a friendly demeanor, and appeared to put forth acceptable effort throughout the two-hour evaluation.

Test results (F1:V.2):

Due to limited availability of Saudi norms at the time of this exam, American norms for PPVT-4, EVT-2, and Bayley Scales were used (with cultural modifications of test and norms).

| **Test** | **Functions Assessed** | **Result/Standard Score (SS)** | **Age equivalent** | **Range/Comments** |
| --- | --- | --- | --- | --- |
| **Leiter International Performance Scale** | Psychometric nonverbal intelligence | 54 | 4:2 years | Her intelligence was assessed as being in the mild to moderate intellectual disability range |
| **McCarthy Scales of Children Abilities**  **Block Design** | Reasoning | 40 | 3:0 years | Working with blocks, She demonstrated nonverbal reasoning skills at the 3:0 year-level |
| **Peabody Picture Vocabulary Test (PPVT-IV)** | Receptive vocabulary | 40 | 3:0 years | Receptive vocabulary was in the moderately impaired range |
| **Expressive Vocabulary Test (EVT-II)** | Expressive vocabulary | 25 | 2:0 years | Expressive vocabulary was more significantly impaired; She was unable to name items such as rabbit, yellow, star, blue, frog, or window |
| **Beery Developmental Test of Visual-Motor Integration (VMI)** | Eye hand integration, visuoconstruction | -- | -- | She was unable to copy any lines or designs (only imitate), thus further fine motor testing below was administered |
| **Bayley III Scale -  Fine Motor** | Fine motor coordination, psychomotor integration, and constructional skills | -- | 3:0 years | Demonstrated a dynamic crayon grasp, mimicked lines in various orientations and a circle; unable to imitate a plus sign, or snip paper with scissors |
| **ADL Screening** | Feeding  Drinking  Dress/undress  Toileting  Instrumental ADLs | --  --  --  --  -- | Independent  Independent  Variable  Independent  Variable | She is able to undress but requires help dressing and bathing. She drinks, feeds, and toilet, independently. She is able to maneuver iPads, and can fetch a snack, but cannot make transactions at grocery store calls or hold a conversation |

Testing thus revealed intellectual skills in the mild to moderate disability level (SS= 54; at the 4:2 year-level). Nearly all tested domains (reasoning, receptive language, and fine motor skills) demonstrated that she mainly functions at the 3:6-year-level. However, expressive language was an area of weakness, placing her in the severely impaired level (SS= 25 at the 2:0 year level). She is fully independent in feeding, ambulating, transferring, and toileting. She requires only minimal supervision in dressing and her instrumental skills of daily living are variable.

The brother’s (F1:V.4) medical assessment:

The patient is the youngest of three siblings. He is a product of full-term pregnancy and cesarean delivery due to a previous C-section. He remained in the NICU for three days due to poor sucking and aspiration. He was discharged with his mother in good condition and has been medically healthy apart from needing myringotomy and adenoidectomy procedures. However, developmental milestones were significantly late with head lag, severe hypotonia, and increased deep tendon reflex. He reportedly crawled at 2 years, and walked at age 3:6 years. Speech currently consists of mostly gesturing and pointing to things he needs. He has not been toilet trained yet.

On medical examination, he was noted to have subtle dysmorphic features (down slanting of the palpebral fissure, carp mouth, and low sitting posteriorly rotated ears), flat feet, and mild hypotonia on neurological examination. Gait appeared unbalanced but normal overall. He was drooling for most of the time. Brain MRI was reportedly read as normal. He received intensive physical therapy at 3 years of age, after which his ambulation significantly improved. He attended a day rehabilitative center at age 4, as well as a similar program the year after. He also received speech and occupational therapy.

His parents are concerned about his poor speech, self-care skills, and very limited and disrupted sleep. They also noted repetitive behaviors and intense fear of loud noises. He does recognize his grandparents and runs to greet them. He reportedly loves cats and runs after them. He can be very impulsive and has a limited understanding of danger. Runs into things while walking. Poor coordination, balance and fine motor skills.

Interview Behavior and Mental Status:

He easily engaged in offered toys. When excited, stereotype behavior was apparent (flapping hands and arms up and down). He did not answer the question "where is your sibling”, or respond to simple playful jokes. Affect was euthymic and cheerful. He smiled appropriately in response to context and demonstrated good joint attention and social reciprocity. He remained seated most of the time and was not hyperactive. Overall, he attempted all tasks presented to him, seemed to enjoy the tasks, and maintained a pleasant demeanor throughout the 2-hour evaluation.

Test Results (F1:V.4):

Due to limited availability of Saudi norms at the time of this testing, American norms were used for the Bayley Cognitive scale, and Vineland Adaptive Behavior Scale, albeit with cultural modifications for tests and norms

| **Test** | **Functions Assessed** | **Standard Score** | **Mental age** | **Range/Comments** |
| --- | --- | --- | --- | --- |
| **Leiter International Performance Scale** | Psychometric, nonverbal intelligence | -- | -- | He did not appear to grasp the concept of this task. As such, the test was discontinued and a formal IQ estimate was not obtained |
| **Bayley III Cognitive Scale** | Cognitive skills | ~ 40 - 55 | 1:6 years | Cognitive function is estimated at the 1:6-year-level. He found hidden objects, removed lid from bottle, and displayed relational play; he did not place shapes in correct slots, or assemble 2-peice puzzles |
| **Bayley III Language Scale** | Receptive  Expressive | --  -- | 1:0 year  1:0 year | Responded to requests of social routines, recognized two words, but did not identify at least one object, or follow 1-part directions. He jabbered expressively, produced consonant-vowel combinations, but not word-approximates |
| **Bayley III Motor Scale** | Fine motor skills  Gross motor skills | --  -- | 2:0 years  1:6 years | Only imitated random strokes via palmar and transitional grasp, and stacked 2 blocks only, and put coins in slots with difficulty. Grossly, he walked up and down stairs, both feet on steps (at times hands as well), with support; he did not run with coordination or kicked ball |
| **Vineland DAILY LIVING SKILLS Subscale** | -- | 45 | 1:7 years | Moderately delayed ADLs for his age |
|  | Personal | -- | 1:9 years | He feeds himself without assistance, is able to undress, but does not pull up garments; he informs mother when he soils himself but is not toilet trained |
|  | Domestic | -- | 1:9 years | At times, puts possessions away when asked |
|  | Community | -- | 1:5 years | Understands danger of hot items, but not the function of money |

Summary, Formulation, and Recommendations:

Although he struggled grasping task requirement of formal IQ tests, he produced an estimated intellect that places him in the moderate mental disability range (~ SS= 40-55), around the 1:6 year-level. Gross motor skills were at a similar level. Fine motor skills and socialization were his strength, at the 2:0-year level, and expressive language was a weakness, at the 1:10 year-level. Self-care is at the 1:7 year-level (able to ambulate independently, self-fed without assistance, drinks and undresses, but does not self-dress and is not toilet trained). He has a stereotyped behavior disorder, common in children with intellectual disability, but displays good social reciprocity and does not have autism.

**Family 2:**

Clinical description:

Three siblings (F2:II.1, F2:II.3, and F2:II.4) who were born to consanguineous parents presented for clinical genetics evaluation due to history of developmental delay and microcephaly.

The eldest affected sibling (F2:II.1) is a 10-year-old female who was born at full term by normal standard vaginal delivery. Mother had concerns of decreased fetal movement during the pregnancy. This sibling was hospitalized at 2 weeks of age for two weeks due to poor feeding. Developmental delay was noticed from a young age. She was able to walk at 2 to 3 years of age. First words were delayed and at age 10 years she has only a few words that she is able to use appropriately. Recent head MRI and EEG were normal. She has no history of seizures. She eats mostly pureed foods due to difficulties with chewing. From a cardiac standpoint, she has a small ventricular septal defect and is monitored by cardiology, but no intervention has been required. On exam at age 10 years 9 months, height is at the 36^th^ percentile, weight at the 11^th^ percentile, and head circumference is 3.5 standard deviation (SD) below the mean. She has bitemporal narrowing, a relatively short forehead, and slightly downslanting palpebral fissures. Teeth appeared slightly large and wide-spaced. She has bilateral 5^th^ finger clinodactyly. Patellar reflexes were normal and she had reasonable muscle strength. She has a mildly ataxic gait.

Sibling F2:II.3 is a 5-year-old male who was born at full-term by Cesarean section due to maternal reasons. Mother noted decreased fetal movement during the pregnancy. Developmental delay was noticed from an early age. He walked at age 2 years. At age 3 years 4 months he had only a few words used appropriately. He has no history of seizures. He eats primarily pureed foods. He was born with a ventricular septal defect that required surgery at age 3 years. On exam at age 3 years 4 months, height is at the 63^rd^ percentile, weight is at the 34^th^ percentile, and head circumference is 3 SD below the mean. He has mild dolichocephaly. Oral exam shows mildly wide-spaced teeth. He has a single palmar crease on the left hand. Patellar reflexes were 1+ bilaterally. He had mild generalized hypotonia.

Sibling F2:II.4 is 2 year-6-month-old male who was born at full term by repeat Cesarean section. He has history of low tone. He was taking independent steps by 2 ½ years of age. He has no words used appropriately. Neurodevelopmental evaluation diagnosed him with autism. Cardiac evaluation has found a patent ductus arteriosus which may need surgical ligation. On exam at age 2 -years-6 months, height is at the 10^th^ percentile, weight is at the 22^nd^ percentile, and head circumference is 3.5 SD below the mean. He has single palmar creases bilaterally. Patellar reflexes were 1+ bilaterally and generalized hypotonia was present.

Chromosomal microarray for the three affected siblings revealed common areas of absence of heterozygosity (AOH) at hg19 coordinates chr20:10,418,800-16,923,134 and chr20:29,448,795-41,483,591.

**Supplemental figures**


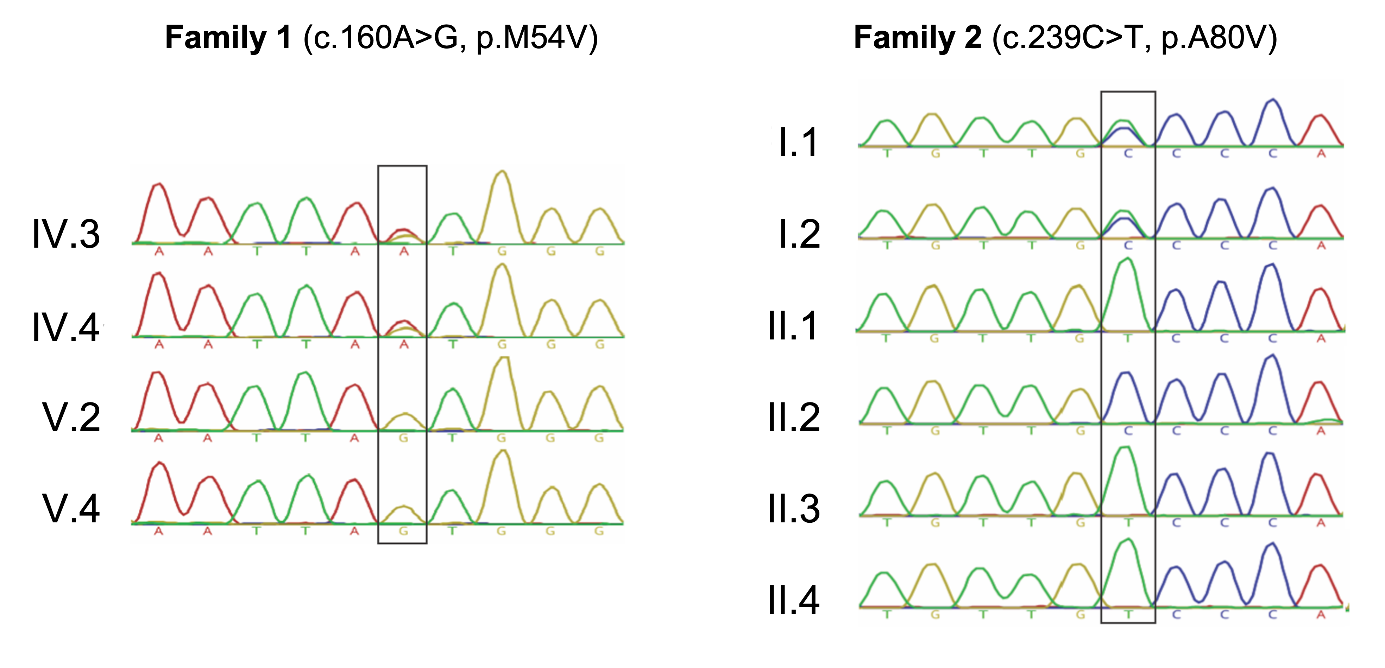


**Fig. S1:** **Rare NAA20 variants identified in two families.** Representative chromatograms are shown for Family 1 *NAA20* variant c.160A>G (p.Met54Val) and Family 2 *NAA20* variant c.239C>T (p.Ala80Val).


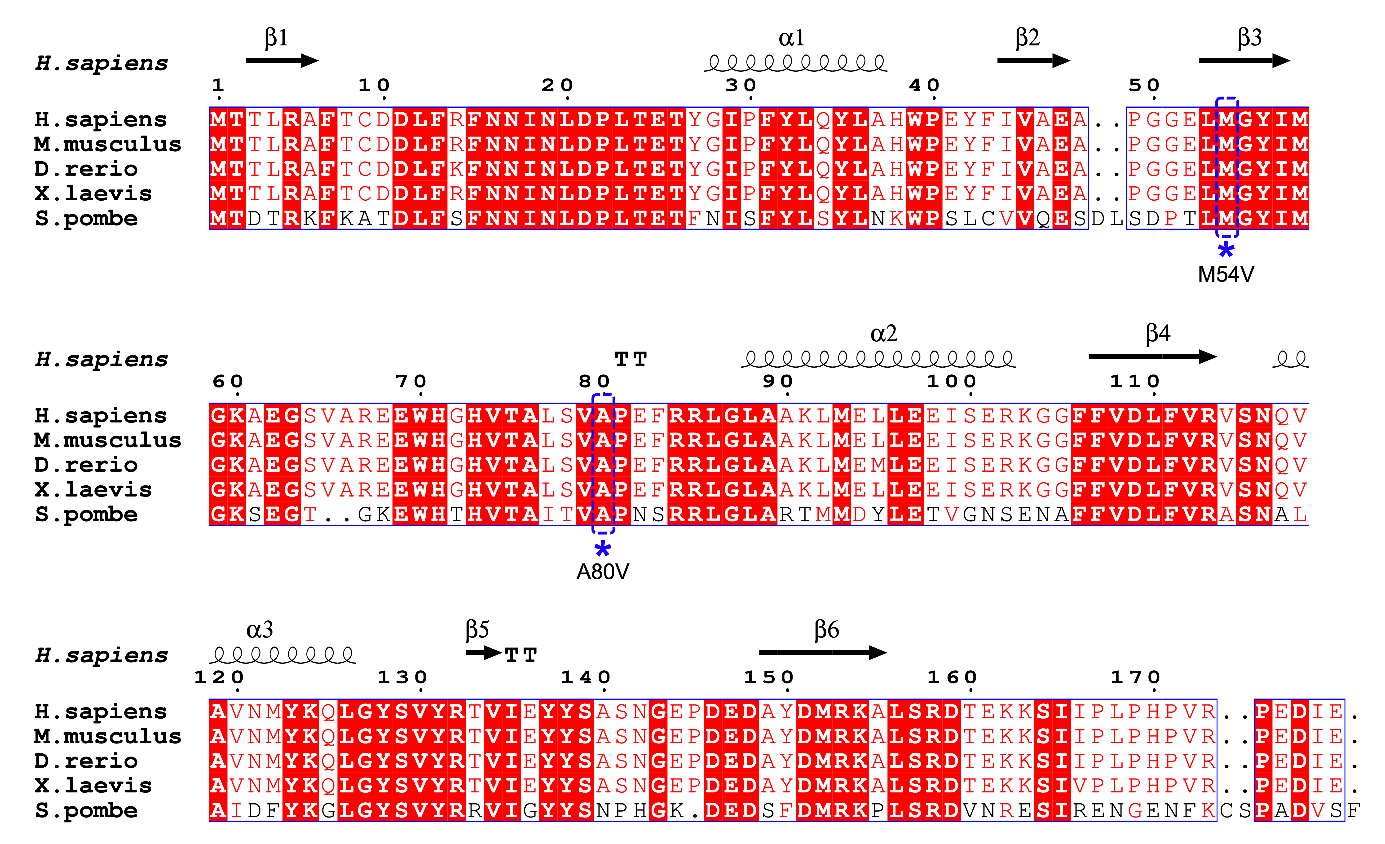


**Fig. S2: NAA20 Met54 and Ala80 are conserved residues.** Sequence alignment of *H. sapiens*, *M. musculus*, *D. rerio*, *X. laevis* and *S. pombe* NAA20 shows the conservation of residues M54 and A80. The positions of the patient variants p.M54 and p.A80 are indicated with blue dashed frames and asterisks.


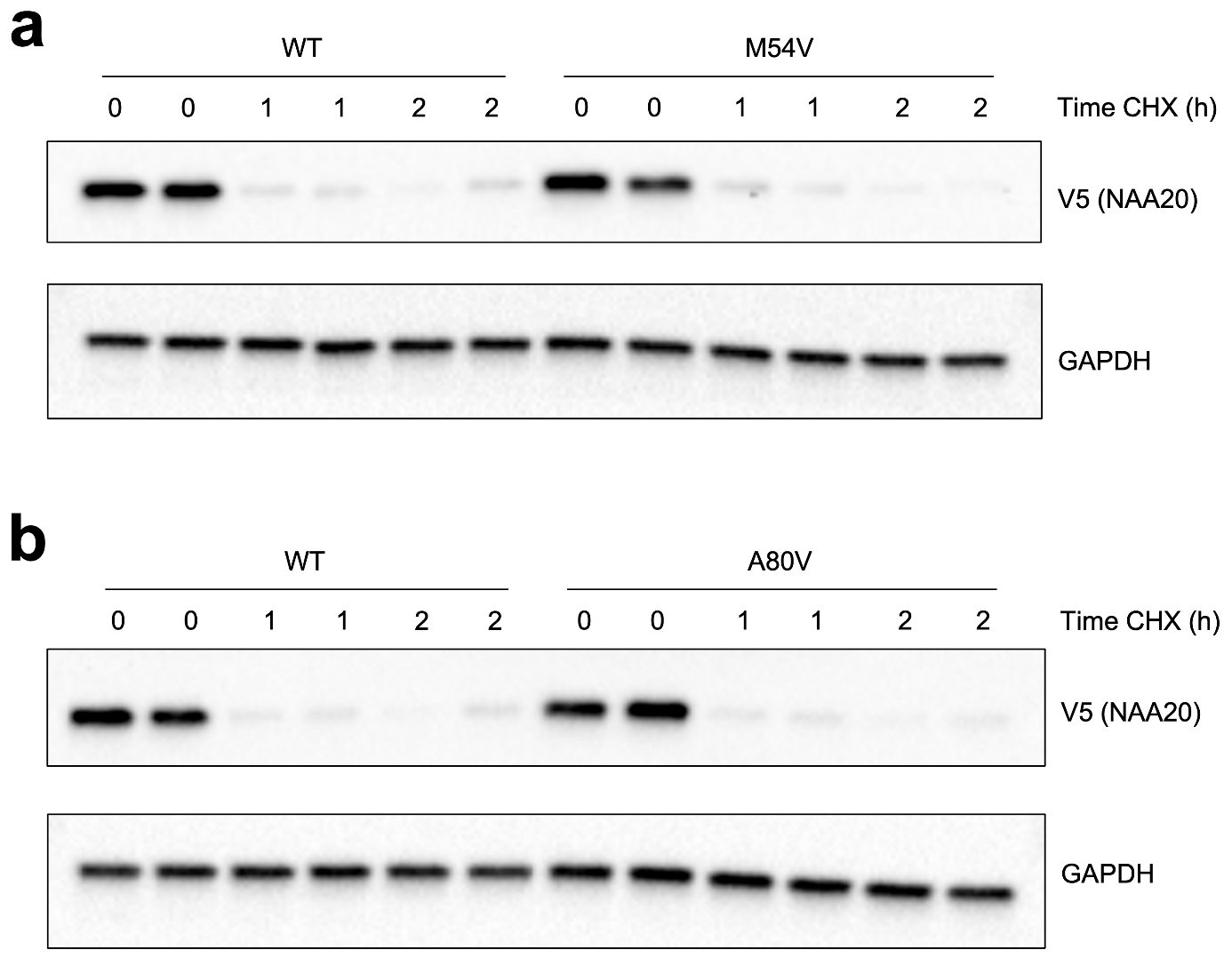


**Fig. S3: Cellular stability of NAA20 M54V and NAA20 A80V.** Cellular stability of NAA20 M54V (a) and NAA20 A80V (b) was assessed by comparative cycloheximide chase assays with NAA20 WT. After transfecting HeLa cells with plasmids expressing NAA20 WT, M54V or A80V, Cycloheximide (CHX) was added for 0, 1 or 2 hours after which cells were harvested, lysed and subjected to Western blotting analysis using anti-V5 (NAA20) and anti-GAPDH as loading control. No significant difference in protein degradation was observed for NAA20 M54V or NAA20 A80V compared to NAA20 WT.


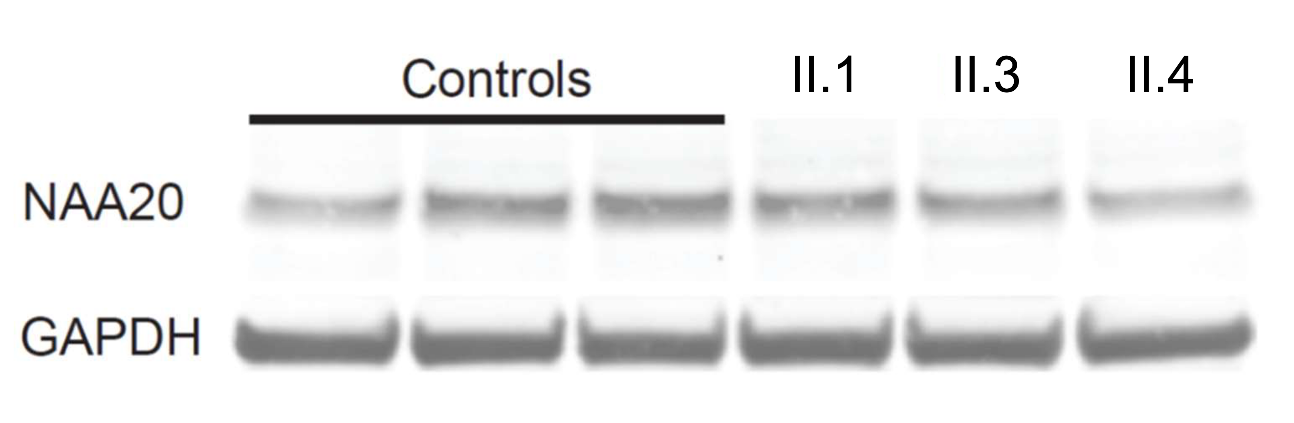


**Fig. S4: Cellular levels of NAA20 in Family 2 lymphoblasts.** Lymphoblasts from all affected individuals of Family 2 (F2:II.1, F2:II.3 and F2:II.4) and three controls were harvested, lysed, and analyzed by SDS-PAGE and Western blotting using anti-NAA20 and anti-GAPDH as loading control. No significant difference in NAA20 protein levels was observed in affected individuals compared to controls.

**Supplemental tables**

**Table S1: *In silico* predictions of *NAA20* variants**

| Variant | NM_016100.4:c.239C>T: p.Ala80Val | | NM_016100.4:c.160A>G: p.Met54Val | |
| --- | --- | --- | --- | --- |
| In Silico tool | **Prediction (where applicable)** | **Score** | **Prediction (where applicable)** | **Score** |
| BayesDel addAF | Damaging | 0.2552 | Damaging | 0.1679 |
| BayesDel noAF | Damaging | 0.1288 | Damaging | 0.003383 |
| CADD | n/a | 28.1 | n/a | 23.3999 |
| DANN | n/a | 0.9992 | n/a | 0.9802 |
| EIGEN | Pathogenic | 0.7646 | Benign | -0.0138 |
| EIGEN PC | Pathogenic | 0.7525 | Benign | 0.1588 |
| FATHMM-MKL | Damaging | 0.9846 | Damaging | 0.9885 |
| FATHMM-XF | Damaging | 0.947 | Damaging | 0.9557 |
| LIST-S2 | Damaging | 0.9893, 0.9898 | Damaging | 0.9668 |
| LRT | Deleterious | 0 | Deleterious | 0 |
| MutPred | Pathogenic | 0.542 | Benign | 0.484 |
| Mutation assessor | Medium | 2.26 | Neutral | -1.71 |
| MutationTaster | Disease causing | 1 | Disease causing | 1 |
| PROVEAN | Damaging | -3.55, -3.58, -3.57 | Damaging | -3.29, -3.53, -3.46 |
| Polyphen2 HDIV | Possibly damaging | 0.854, 0.925 | Benign | 0.02, 0.006 |
| Polyphen2 HVAR | Possibly damaging | 0.706 | Benign | 0.1, 0.062 |
| PrimateAI | Damaging | 0.8975 | Damaging | 0.8682 |
| SIFT | Damaging | 0.002, 0.001 | Tolerated, Damaging | 0.087, 0.044, 0.088 |
| DEOGEN2 | Tolerated | 0.07362, 0.1878 | Tolerated | 0.1973, 0.08915 |
| FATHMM | Tolerated | 1.84 | Tolerated | 2.49 |
| MVP | Benign | 0.7613 | Benign | 0.3048 |
| MetaLR | Tolerated | 0.1794 | Tolerated | 0.0229 |
| MetaSVM | Tolerated | -0.7701 | Tolerated | -0.9454 |
| REVEL | Benign | 0.569 | Benign | 0.328 |
| SIFT4G | Tolerated, Damaging | 0.057, 0.068, 0.001 | Tolerated | 0.483, 0.268, 0.477 |
| PhastCons100way | n/a | 1.000 | n/a | 1.000 |
| PhyloP100way | n/a | 7.806 | n/a | 9.023 |
| GERP | n/a | 5.3699 | n/a | 4.6199 |
| PhyloP17way (primate) | n/a | 0.5989 | n/a | 0.7559 |
| PhyloP30way (mammalian) | n/a | 1.026 | n/a | 1.312 |
| PhyloP100way (vertebrate) | n/a | 7.893 | n/a | 9.076 |
| PhastCons17way (primate) | n/a | 0.994 | n/a | 0.971 |
| PhastCons30way (mammalian) | n/a | 0.99 | n/a | 0.9959 |
| PhastCons100way (vertebrate) | n/a | 1 | n/a | 1 |
| fitCons-gm | n/a | 0.7248 | n/a | 0.7248 |
| fitCons H1 (H1-hESC) | n/a | 0.6435 | n/a | 0.7097 |
| fitCons HU (HUVEC) | n/a | 0.7144 | n/a | 0.6133 |
| Integrated fitCons | n/a | 0.7065 | n/a | 0.7063 |
| SiPhy29way | n/a | 17.8745 | n/a | 13.1241 |
| MPC | n/a | 1.3338 | n/a | 0.5587 |
| Bstatistic | n/a | 672 | n/a | 648 |

**Table S2: Summary of clinical findings in five affected individuals with recessive *NAA20* missense variants**

| **Family** | Family 1 | | Family 2 | | |
| --- | --- | --- | --- | --- | --- |
| **Individual** | F1:V.2 | F1:V.4 | F2:II.1 | F2:II.3 | F2:II.4 |
| **Gender** | female | male | female | male | Male |
| **Age at most recent examination** | 13 years | 7 years | 10 years 9 months | 3 years 4 months | 2 years 6 months |
| **Ethnicity** | Saudi Arabian | Saudi Arabian | Iraqi | Iraqi | Iraqi |
| ***NAA20* variants NM_016100.4** | c.160A>G (p.Met54Val) | c.160A>G (p.Met54Val) | c.239C>T (p.Ala80Val) | c.239C>T (p.Ala80Val) | c.239C>T (p.Ala80Val) |
| **Variant type** | homozygous | homozygous | homozygous | homozygous | homozygous |
| **Body height and weight** | weight and height at 75th percentile | At age 2.3 years, height is at the 86th percentile, weight at 63rd percentile. | At age 10 years 9 months, height is at the 36^th^ percentile, weight at the 11^th^ percentile | At age 3 years 4 months, height is at the 63^rd^ percentile, weight is at the 34^th^ percentile | At age 2 -years-6 months, height is at the 10^th^ percentile, weight is at the 22^nd^ percentile |
| **Head circumference** | -2.3 SD | -1.9 SD | <1st % (-3.5 SD) | <1st % (-3.0 SD) | <1st % (-3.5 SD |
| **Facial and other dysmorphisms** | Subtle dysmorphic features; prominent philtrum; thick upper lips and epicanthal folds | Subtle dysmorphic features; down slanting of the palpebral fissure; carp mouth; and low sitting posteriorly rotated ears | Bitemporal narrowing; short forehead; downslanting palpebral fissures; large and wide-spaced teeth; b/l 5^th^ finger clinodactyly | Mild dolichocephaly; wide-spaced teeth; u/l single palmar crease | B/l single palmar creases |
| **Ambulation** | Walked at age 2.5 years. Gait and overall motor function were unremarkable, reduced fine motor skills | Walked at age 3.5 years. Poor coordination, balance and fine motor skills. | Walked at age 2-3 years | Walked at age 2 years | Walked at age 2.5 years |
| **Speech development** | First words at age 4-5 years. Impaired vocabulary. | Very limited. Mostly gesturing. | First words were delayed and at age 10 years she has only a few words used appropriately | At age 3 years 4 months he had only a few words used appropriately. | He has no words used appropriately |
| **Neurodevelopmental** | Mild to moderate ID.  Subtle dysmorphic autistic features.  (SS= 54; at the 4:2 year level at 8:9 years of age) | Moderate ID. Not autism  (~ SS= 40-55; around 1:6 year level at 5:2 years of age) | ID | ID | ID Autism |
| **Seizures** | no | no | no | no | No |
| **Cardio** | normal | normal | small ventricular septal defect | ventricular septal defect requiring surgery | patent ductus arteriosus |
| **Other** | Brain MRI revealed patchy foci of increasing signal intensity seen in the bilateral frontal subcortical region | - | Brain MRI: normal. Appears to be low craniofacial ratio, compatible with  microcephaly. | Brain MRI: Corpus callosum is short and appears comma shape with somewhat truncated  splenium. Myelination appears delayed for age. | Brain MRI: Symmetric prominence of extra-axial CSF spaces in bilateral frontal and  temporal regions, likely representing benign enlargement of subarachnoid  space. This is within normal limits for patient's age. |

**Table S3** – excel file

**Supplemental Materials and methods**

**DNA analysis – Exome sequencing and genome sequencing**

Genomic DNA was obtained from peripheral blood. For Family 1, exome sequencing was performed on both affected siblings (F1:V.2 and F1:V.4) at King Faisal Specialist Hospital and Research Centre’s genomic core facility. DNA libraries were prepared using an Agilent Sureselect All Exons V5 (50 Mb) capture kit (Agilent Technologies; Santa Clara, Ca, USA). Genomic DNA was mechanically fragmented and captured by probe hybridisation prior to amplification and sequencing on Illumina HiSeq2500 (Illumina Inc; San Diego, Ca, USA) for paired-end 100nt sequencing. Sequence alignment, indexing of the reference genome (hg19), variant calling and annotation used a pipeline based on BWA, Samtools, GATK (<https://software.broadinstitute.org/gatk/>) and Annovar, respectively. Variant annotation was performed using a combination of available public knowledge databases provided by the Annovar package and in-house databases which include collections of previously published Saudi disease causing variants. Autozygome mapping was performed as previously described by ^1^. For Family 2, genome sequencing was performed on the three affected children (F2:II.1, F2:II.3 and F2:II.4) and both parents (F2:I.1 and F2:I.2) at Genewiz (South Plainfield, NJ, USA). Genomic DNAs were fragmented, and DNA libraries were prepared using the Illumina TruSeq Nano kit, and sequenced on Illumina HiSeq X series (Illumina, San Diego, CA, USA) with 150-bp, paired-end reads. Data analysis was performed using Illumina HiSeq Analysis Software v2.2. The five samples had 93.04-94.03% of bases at ≥30X coverage. Variants were filtered with Qiagen Clinical Insight (Qiagen, Redwood City, CA, USA) based on confidence, frequency (≤0.2% allele frequency in subpopulation with highest frequency in gnomAD, ExAC, NHLBI ESP exomes, and 1000 Genomes Project), and predicted deleteriousness (coding or splicing change or CADD score ≥20 included). Homozygous variants were prioritized given consanguinity. This filtering strategy resulted in the identification of 9 variants (Table S3). The identified NAA20 c.239C>T;p.A80V was confirmed by Sanger sequencing to be heterozygous in the parents and homozygous in the three affected children. The unaffected sibling (F2:II.2) was also sequenced and was noted to be homozygous wild-type.

**Multiple sequence alignment and structural modelling**

A multiple sequence alignment was generated using Clustal Omega ^2^ and illustrated by ESPript 3.0 ^3^. Structural analysis of human NatB (PDB ID: 6VP9) ^4^ was performed using PyMOL (Schrödinger, LLC The PyMOL Molecular Graphics System, Version 2.3. 2019).

**Construction of variant NAA20 plasmids**

Vectors expressing NAA20-M54V and NAA20-A80V were generated using Q5 site directed Mutagenesis Kit (New England Biolabs; MA, USA) and pcDNA 3.1 NAA20-V5-His vector as template ^5^. The primers used for NAA20 M54V were 5´-TGGAGAATTAGTGGGTTATATTATGGGTAAAG-3´ and 5´-CCAGGTGCCTCTGCAACA-3´ with annealing temperature 65°C, and for NAA20 A80V 5´-CTGTCTGTTGtCCCAGAATTTCGAC-3´and 5´-AGCTGTGACGTGCCCGTG-3´ with annealing temperature 67°C. The plasmid variants were verified by DNA-sequencing.

**Expression and Immunoprecipitation of NAA20-WT and variants**

Cells were grown for 24 hours before transfection (1,500,000 cells/10 cm dish for HeLa cells, ATCC, CCL-2, routinely tested for mycoplasma infection, authenticated upon receipt). Transfection was performed using XtremeGENE 9 Transfection reagent (Roche, Basel, Switzerland) and 4 µg of plasmid DNA. After 24 h, transfected cells were washed in cold PBS, harvested in cold PBS by centrifugation 17,000 x *g* for 1 min and frozen at -80°C.

For immunoprecipitation (IP), pellets were thawed, resuspended in lPH lysis buffer (50 mM Tris-HCl pH 8.0, 150 mM NaCl, 5 mM EDTA, 0.5 % NP40, Complete EDTA free protease inhibitor (Roche), 200 ml IPH per dish) and tubes were left at 4°C on a rotator at for 25 min. The lysates were centrifuged at 15,600 x *g* for 1 min and 1 µg V5-tag antibody (Invitrogen #R960-25, CA, USA)/ dish was added to the supernatant. The mixture was incubated at 4°C on a rotator for 3h before 17 µl prewashed magnetic beads (Invitrogen Dynabeads 10004D)/dish were added and again incubated at 4°C on a rotator overnight. Beads were washed three times in IPH lysis buffer and one time in 2x acetylation buffer (100 mM Tris-HCl (pH 8.5), 20% Glycerol, 2 mM EDTA) and used in a [14C]-Ac-CoA–based acetylation assay.

**[^14^C]-Ac-CoA–Based Acetylation Assay**

Magnetic beads from IP (10 µl), [^14^C]-Ac-CoA (84 μM), Ac-CoA (116 μM) and 200 μM NatB substrate peptide MDEL (derived from the N-terminus of NF-kκB p65, Q04206, peptide purity >95%, Innovagen, custom made): [H] MDELFPL*RWGRPVGRRRRPVRVYP* [OH] ^5^ or variants in amino acid residue position two resembling the other major substrate types of NatB [H] MEELFPL*RWGRPVGRRRRPVRVYP* [OH], [H] MNELFPL*RWGRPVGRRRRPVRVYP* [OH], or [H] MQELFPL*RWGRPVGRRRRPVRVYP* [OH] were mixed in acetylation buffer (50 mM Tris-HCl (pH 8.5), 10% Glycerol, 1 mM EDTA) to a final volume of 30 µl and incubated at 37°C on a shaker for 2 h. Beads were isolated using a magnet, and 20 μl of the suspension was transferred to a P81 Phosphocellulose paper. After airdrying filters, the filters were washed 3 x 5 min in Hepes pH 7.4 and again airdried. To determine the amount of incorporated [14C]-Ac, the filter discs were added to 5 ml Ultima Gold F scintillation mixture (Perkin-Elmer) and analysed by a Perkin-Elmer TriCarb 2900TR Liquid Scintillation Analyzer. For each condition three measurements were performed. For each sample, IP with cells derived from 3 x 10 cm dishes were used. Three independent experiments were performed. Data are presented as mean +/- s.d. A Two-sided Student t-test with the most stringent condition (assumed unequal variance) was used to assess significance.

**Cycloheximide chase assay**

HeLa cells were grown for 20 hours before transfection (250,000 cells/well in a 6-well tray). Transfection was performed using XtremeGENE 9 Transfection reagent (Roche, Basel, Switzerland) and 0.7 µg of plasmid DNA per well (2 wells per plasmid per timepoint). After 36 h, transfected cells were added Cycloheximide (CHX) 50 μg/ml (Calbiochem, 239765) to arrest translation and harvested 0, 1, and 2 hours post CHX addition in cold PBS by centrifugation 17,000 x *g* for 1 min. After lysis in IPH buffer, samples were analyzed by SDS-PAGE and Western blotting using 7 µg total protein loaded /well in a 15 well BioRad gel setup.

**Western Blot Analysis**

Cell lysates and IP samples were analyzed by Western blotting. Proteins were separated by SDS-PAGE and transferred onto a nitrocellulose membrane (Amersham Protran 0.2 µM NC) by immunoblotting. The membrane was blocked in 5% non-fat dry milk and incubated overnight with anti-V5 antibody (Invitrogen #R960-25, CA, USA) diluted 1:10 000, NAA25 antibody (Sigma Aldrich, HPA039322) diluted 1:300, NAA20 antibody (Sigma Aldrich, HPA063344) or anti-GAPDH antibody (Santa Cruz Biotechnology, FL-335, sc-25778) diluted 1:3000 in 1xPBS containing 1% non-fat dry milk and 0.1% Tween. The immunoblots were washed and incubated for 1 h at room temperature with HRP-linked secondary antibody donkey anti-rabbit IgG (GE Healthcare, NA934) or HRP-linked secondary antibody sheep anti-mouse IgG (GE Healthcare, NA931). The HRP-signal was detected using SuperSignal^TM^ West Pico PLUS Chemiluminescent Substrate Kit (Thermo Scientific). The ChemiDoc^TM^ XRS+ system (Bio-Rad, CA, USA) coupled with Image Lab^TM^ Software (Bio-Rad, CA, USA) was used for visualization and quantification of protein bands. Immunoprecipitation experiments (n=9) were analyzed by a two-tailed Student t-test with unequal variance to assess significance. Data are presented as mean +/- s.d.

**Supplemental references**

1. Carr, I.M., Bhaskar, S., O'Sullivan, J., Aldahmesh, M.A., Shamseldin, H.E., Markham, A.F., Bonthron, D.T., Black, G., and Alkuraya, F.S. (2013). Autozygosity mapping with exome sequence data. Hum Mutat *34*, 50-56. 10.1002/humu.22220.

2. Sievers, F., Wilm, A., Dineen, D., Gibson, T.J., Karplus, K., Li, W., Lopez, R., McWilliam, H., Remmert, M., Soding, J., et al. (2011). Fast, scalable generation of high-quality protein multiple sequence alignments using Clustal Omega. Mol Syst Biol *7*, 539. 10.1038/msb.2011.75.

3. Robert, X., and Gouet, P. (2014). Deciphering key features in protein structures with the new ENDscript server. Nucleic Acids Res *42*, W320-324. 10.1093/nar/gku316.

4. Deng, S., Pan, B., Gottlieb, L., Petersson, E.J., and Marmorstein, R. (2020). Molecular basis for N-terminal alpha-synuclein acetylation by human NatB. Elife *9*. 10.7554/eLife.57491.

5. Starheim, K.K., Arnesen, T., Gromyko, D., Ryningen, A., Varhaug, J.E., and Lillehaug, J.R. (2008). Identification of the human N(alpha)-acetyltransferase complex B (hNatB): a complex important for cell-cycle progression. Biochem J *415*, 325-331. 10.1042/BJ20080658.
